# Factors influencing elderly women's mammography screening decisions: implications for counseling

**DOI:** 10.1186/1471-2318-7-26

**Published:** 2007-11-16

**Authors:** Mara A Schonberg, Ellen P McCarthy, Meghan York, Roger B Davis, Edward R Marcantonio

**Affiliations:** 1 Division of General Medicine and Primary Care, Department of Medicine, Harvard Medical School, Beth Israel Deaconess Medical Center, Boston, MA, USA

## Abstract

**Background:**

Although guidelines recommend that clinicians consider life expectancy before screening older women for breast cancer, many older women with limited life expectancies are screened. We aimed to identify factors important to mammography screening decisions among women aged 80 and older compared to women aged 65–79.

**Methods:**

Telephone surveys of 107 women aged 80+ and 93 women aged 65–79 randomly selected from one academic primary care practice who were able to communicate in English (60% response rate). The survey addressed the following factors in regards to older women's mammography screening decisions: perceived importance of a history of breast disease, family history of breast cancer, doctor's recommendations, habit, reassurance, previous experience, mailed reminder cards, family/friend's recommendations or experience with breast cancer, age, health, and media. The survey also assessed older women's preferred role in decision making around mammography screening.

**Results:**

Of the 200 women, 65.5% were non-Hispanic white and 82.8% were in good to excellent health. Most (81.3%) had undergone mammography in the past 2 years. Regardless of age, older women ranked doctor's recommendations as the most important factor influencing their decision to get screened. Habit and reassurance were the next two highly ranked factors influencing older women to get screened. Among women who did not get screened, women aged 80 and older ranked age and doctor's counseling as the most influential factors and women aged 65–79 ranked a previous negative experience with mammography as the most important factor. There were no significant differences in preferred role in decision-making around mammography screening by age, however, most women in both age groups preferred to make the final decision on their own (46.6% of women aged 80+ and 50.5% of women aged 65–79).

**Conclusion:**

While a doctor's recommendation is the most important factor influencing elderly women's mammography screening decisions, habit and reassurance also strongly influence decision-making. Interventions aimed at improving clinician counseling about mammography, which include discussions around habit and reassurance, may result in better decision-making.

## Background

There is great heterogeneity in health among older women leading to substantial differences in life expectancy [1]. For instance, women aged 80–84 in the top quartile of health have 13 years of life expectancy while women aged 80–84 in the lowest quartile of health have only 4.6 years of life expectancy [1]. Meanwhile, experts generally agree that a woman needs 5 to even 10 years of life expectancy to potentially benefit from mammography screening [1,2]. Benefits of mammography screening among older women include possibly prolonging life or preventing morbidity associated with advanced breast cancer [3,4]. However, potential risks include complications and anxiety related to finding and treating breast cancers that would never have become clinically significant in an older woman's lifespan [1]. Therefore, guidelines recommend that clinicians consider older women's life expectancy and comorbidities before recommending mammography screening [5,6]. Increasingly more women aged 80 and older are undergoing mammography screening and evidence suggests it is not being targeted to the oldest women in the best health and most likely to benefit [7,8].

To better understand elderly women's mammography screening decisions, we interviewed women aged 80 and older and physicians who cared for these women using qualitative methods [9]. In that study, we developed a conceptual framework of the factors that influence mammography screening decisions of women aged 80 and older, including: 1) Patient factors (e.g., risk perception, habit, history of breast disease, etc.); 2) System factors (e.g., access, mailed reminders); 3) Social Influences (e.g., daughter's encouragement, family/friends' experience with breast cancer); and 4) Physician influences. We also found that physicians feel uncomfortable discussing stopping screening with women aged 80 and older. Since qualitative data cannot be used to determine the prevalence of attitudes or beliefs in a population and since qualitative methods do not allow for statistical comparisons between groups, we designed a telelphone survey to determine which factors identified in our qualitative study were most important to older women's mammography screening decisions. We were most interested in factors that influence elderly women to choose screening since these factors may need to be addressed before elderly women can feel comfortable stopping screening. We compared responses for women aged 80 and older with women aged 65–79 to see if certain factors need to be specifically addressed among the oldest women. In addition, we examined whether older women preferred that their physician make the decision whether or not they should get sreened or whether they preferred to make the decision on their own or share the decision with their physician.

## Methods

### Study Sample

We telephone surveyed English speaking women aged 65 or older that received their primary care at a hospital based general internal medicine practice in Boston to learn about their mammography screening decisions. The practice consists of approximately 50 faculty internists, over 100 internal medicine residents, and 10 nurse practitioners that provide care to approximately 34,000 patients. We excluded patients who had a history of dementia, significant hearing loss, or were terminally ill as determined by chart review and/or by patients' primary care physicians. We also excluded patients whose physicians thought that answering survey questions would be too psychologically disturbing (e.g., patient had just lost a spouse or was mentally ill). Our initial electronic search identified 716 women aged 80 and older and 1,962 women aged 65–79 who had at least one primary care billing record from a clinic visit in the past year. Since we anticipated greater exclusion criteria among women aged 80 and older, we randomly identified 400 women aged 80 and older and 275 women aged 65–79 from these lists to reach a targeted sample of 200 women (100 per age group). We obtained consent from each patient's primary care physician and we sent women deemed eligible a letter informing them of the study with an opt-out card. The 30-minute survey was administered by one of two study investigators (MS, MY) or by one research assistant. The institutional review board at the Beth Israel Deaconess Medical Center approved our study.

### Data Collection

Our survey first asked women whether or not they had received a screening mammogram in the past two years. We then asked women who had undergone mammography screening in the past two years how important each of the following factors (identified in our qualitative study) were in their screening decision, including: history of breast disease (if applicable), family history of breast cancer (if applicable), a doctor's recommendation, habit (meaning that the woman always got a mammogram every year or so), reassurance (meaning that a normal mammogram would reassure a woman about her health), a mailed reminder card, a family member's recommendation, a friend's recommendation, a friend's experience with breast cancer, age, health, and the media [9]. We asked women who had not undergone mammography screening in the past two years how important their previous experience with mammography, doctor's counseling, habit, health, and the media were in their decision not to get a mammogram. Women rated the influence of each factor on their mammography screening decision on a 4-point scale (essential, very important, somewhat important or not at all important to their decision) [10]. After evaluating each measure individually, we then asked women to rank from 1 to 3, in order of importance, the factors that most influenced their decision to get a mammogram or not to get a mammogram. We also asked women who did not undergo mammography screening recently how strongly they agreed with the statement "I am not concerned about breast cancer" on a 5-point Likert scale. Women could rank lack of concern about breast cancer as one of their reasons for choosing not to be screened.

In addition, we asked women their preferred role in decision-making around mammography screening using a scale created by Degner et al [11]. Responses were categorized into 3 groups: the patient prefers to make her own decision about mammography screening, the patient prefers her doctor makes the decision, or the patient prefers that she share the decision with her doctor. We additionally obtained data on patient's race/ethnicity, education, income, marital status, functional dependency, and perceived health status [12]. We pre-tested the survey on 10 women who were identified using the methods above and we amended the survey based on these interviews.

To compare respondents with non-respondents we obtained data on patients' insurance, race/ethnicity, illness burden, and receipt of mammography in the past 2 years, from patients' medical records. We reviewed one year of patient clinic notes and their current problem list to collect data on patients' illness burden to calculate a Charlson Comorbidity Index (CCI) [13].

### Statistical Analyses

Chi-square statistics were used to compare race/ethnicity, education, insurance coverage, income, perceived health status, CCI, and functional dependency between women aged 80 and older and women aged 65–79. For analyses involving participants we only used data collected from telephone interviews for consistency. Chi-square statistics were also used to compare respondent characteristics with non-respondents. We additionally used chi-square statistics to compare the relative importance of different factors (habit, reassurance, history of breast disease, reminder cards, doctor's recommendations, family history, health, friend's experience with breast cancer, age, family member recommendation, friend recommendation, and the media) on older women's decisions to undergo mammography screening and to compare the relative importance of different factors (habit, doctor counseling, age, previous experience with mammography, health, media, and no concern about breast cancer) on older women's decisions not to be screened. To construct a list of factors influencing older women's screening decision by age and order of importance, we weighted whether a woman ranked a factor as 1, 2, or 3 and then averaged the score provided to each factor across all women within the age group (first choices were scored as 3 and the third choices were scored as 1). We also used the two sample t-test and/or the Wilcoxon Rank Sum Test to compare whether the score calculated for each factor differed significantly by age. Finally, we compared older women's preferred role in decision-making around mammography screening with younger women and we compared receipt of mammography screening by women's preferred role in decision-making. All statistical analyses were performed using SAS statistical software, version 9.1 (SAS Institute, Cary, NC).

## Results

Of the 166 women aged 80 and older and 170 women aged 65–79 ultimately eligible for our study, 102 women aged 80 and older and 98 women aged 65–79 agreed to be interviewed, resulting in a combined response rate of 60%. Non-respondents (n = 136) were similar to respondents with regard to age, race/ethnicity, insurance, CCI, number of clinic visits in the past year, and receipt of mammography. Five women initially identified in the 65–79 age group had turned 80 years by the time they were interviewed and their responses were included with women aged 80 and older. Of the 339 women excluded from the study, 115 did not speak English, 82 had dementia, 77 had left the practice, 24 were deceased, 17 were hearing impaired, 15 were terminally ill, and 9 had physicians who thought the survey would be too psychologically disturbing.

Of the 200 women who participated in the survey, 53.5% were aged 80 and older (mean age 85.3 years) and 46.5% were aged 65–79 (mean age 71.5 years). The majority were non-Hispanic white (65.5%) and were in good to excellent health (82.8%). Most (81.3%) had undergone mammography screening in the previous 2 years (88.2% of women aged 65–79 and 75.2% of women aged 80 and older, p = 0.02). Women aged 80 and older were significantly more likely than women aged 65–79 to report a household income less than $20,000 per year (55.7% vs. 28.8%) and to have a functional dependency (47.7% vs. 14.0%) (Table 1).

**Table 1:** Characteristics of respondents by age group.

		Age Group		
		65–79	80+	p value
		(n = 93)	(n = 107)	
		%	%	
Race:	Non-Hispanic White	60.2	66.4	0.35
	Non-Hispanic Black	30.1	29.0	
	Other	9.7	4.7	
Education:	< High School	16.1	14.2	0.39
	High School Graduate	28.0	35.9	
	Some College	28.0	18.9	
	College Graduate or beyond	28.0	31.1	
Income: (n = 168)	<$20K	28.8	55.7	<0.01
	$20K-<35K	21.2	15.9	
	$35K or more	50.0	28.4	
Insurance:	Private	78.5	85.1	0.23
	Other	21.5	14.9	
Marital Status:	Married	43.0	17.8	<.01
	Widowed	22.6	60.8	
	Other	34.4	21.5	
Clinic Visits in past year:	1	12.9	11.2	0.74
	2–4	49.5	45.8	
	5+	37.6	43.0	
CCI:	0	49.5	43.9	0.15
	1	29.0	22.4	
	2+	21.5	33.6	
Function:	None	86.0	52.3	<0.01
	IADL dependency only*	12.9	37.4	
	ADL dependency*	1.1	10.3	
Perceived Health: (n = 192)	Excellent	31.2	18.2	0.08
	Very Good	26.9	30.3	
	Good	29.0	30.3	
	Fair	8.6	19.2	
	Poor	4.3	2.0	
Screening Mammogram in the past 2 years		88.2	75.2	0.02

* ADL = Activity of Daily Living, IADL = Instrumental Activity of Daily Living

Table 2 illustrates the proportion of women in each age group that considered various factors essential/very important to their mammography screening decision. Although there were no statistical differences by age, among women who were recently screened (n = 162) the majority considered a history of breast disease, a doctor's recommendation, receipt of a reminder card, reassurance, and habit as essential/very important factors in their decision (Table 2). Fewer women considered family history of breast cancer, family member or friend's recommendation, friend's experience with breast cancer, health, age, or the media as essential/very important to their decision. Only small numbers of older women chose not to be screened and there were no statistical differences in factors influencing this decision by age (Table 3). However, the majority of women aged 80 and older considered age as essential/very important to their decision not to be screened. In addition, 80.8% of women aged 65 and older who chose not to be screened were not concerned about breast cancer.

**Table 2:** Proportion of women who considered each factor essential/very important in decision to get a mammogram.

	65–79 (n = 82)	80+ (n = 80)	p Value
Habit	76.2	87.0	0.08
Reassurance	81.0	73.0	0.23
History of Breast Disease	65.0	85.7	0.09
(n = 20 aged 65–79 and n = 28 aged 80+)			
Reminder Card	72.6	73.7	0.90
(n = 62 aged 65–79 and n = 38 aged 80+)			
MD Recommendations	60.2	66.2	0.43
Family History	42.3	38.1	0.77
Health	36.6	47.1	0.19
Friend's Experience with Breast Cancer	30.8	25.0	0.57
Age	28.2	29.0	0.92
Family Member Recommendation	15.9	13.9	0.73
Friend Recommendation	16.9	6.3	0.06
Media	15.5	16.0	0.93

**Table 3:** Proportion of women who considered each factor essential/very important in decision NOT to get a mammogram.

	65–79 (n = 11)	80+ (n = 26)	P Value
	% (proportion n's)	% (proportion n's)	
Habit	50.0% (3/6)	40.0% (8/20)	0.66
MD Counseling	28.6% (2/7)	50.0% (11/22)	0.32
Age	50.0% (3/6)	56.5% (13/23)	0.77
Previous Experience with Mammography	28.6% (2/7)	18.8% (3/16)	0.60
Health	50.0% (3/6)	19.1% (4/21)	0.13
Media	0% (0/6)	8.3% (2/24)	0.46
Not Concerned about Breast Cancer	83.3% (5/6)	80.0% (16/20)	0.86

**Figure 1:**
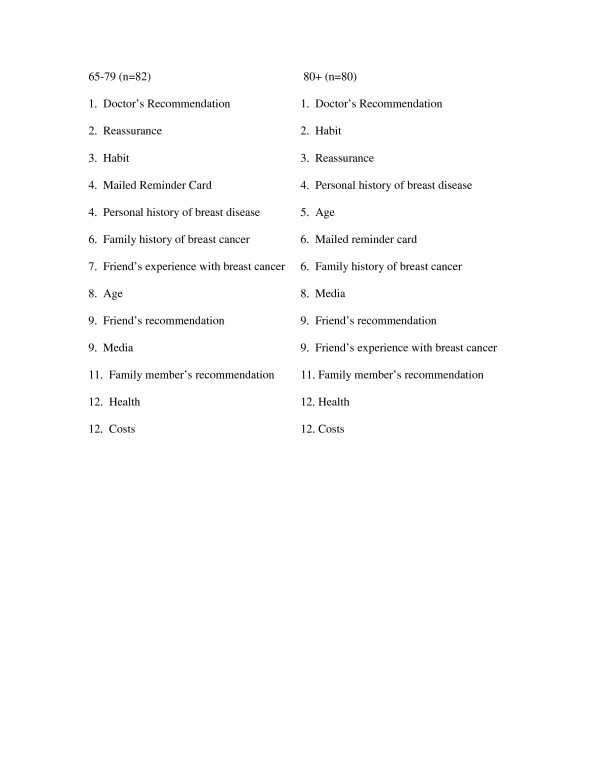
How older women ranked factors influencing their decision to undergo mammography screening in the past two years.* * To create these rankings we asked women to rate the 3 factors most influential to their decision to undergo mammography in the past 2 years. We then weighted whether a woman ranked a factor as 1, 2, or 3 and averaged the score given to each factor across all women. We listed the factors by highest average score to lowest average score.

Figure 1 demonstrates how older women ranked the importance of different factors on their decision to undergo mammography screening. Both women aged 65–79 and women aged 80 and older ranked their doctor's recommendation as the most important factor influencing their decision. Habit and reassurance were the next two factors influencing older women's decision to get screened. There were no significant differences in scores given to factors that influence older women's mammography screening decisions by age. Women aged 80 and older who did not get screened with mammography in the past two years ranked age and then doctor's counseling as the most important factors influencing this decision. Women aged 65–79 ranked a previous negative experience with mammography as the most important factor influencing their decision not to get screened and then a lack of concern about breast cancer. Women aged 80 and older were significantly more likely than younger women to score age highly as a factor influencing their decision not to get screened (p = 0.02).

Table 4 demonstrates receipt of mammography screening among older women by their preferred role in decision-making around screening and by their age. There were no statistically significant differences in women's preferred role in decision-making by age. However, most women in both age groups preferred to make the final decision on their own about whether or not to undergo mammography screening. Women aged 80 and older who preferred to share their mammography screening decision with their doctor were less likely to be screened (59.1%) than women aged 65–79 who preferred to share their decision with their doctor (96.2%). In addition, in post hoc analyses using the Fischer Exact test, we found that women aged 80 and older who preferred to share their decision and perceived themselves to be in good to excellent health were significantly more likely to be screened (76.5%, n = 13/17) than those who preferred to share their decision and perceived themselves to be in fair or poor health (0% n = 0/4) (p = 0.01). Receipt of screening did not differ by perceived health among women aged 80 and older who did not prefer to share their decision making around screening with their physician.

**Table 4:** Receipt of mammography screening in the past 2 years by preferred role in decision-making around screening and by age.

	Women aged 65–79 (n = 93)		Women aged 80 and Older (n = 103)		
	Overall	(Proportion who reported being screened)	Overall	(Proportion who reported being screened)	P value*
Preferred Role in Decision Making:*					
Doctor makes the final decision	21.5% (n = 20)	(19/20 or 95.0%)	32.0% (n = 33)	(27/32† or 84.8%)	0.25
Patient makes the final decision	50.5% (n = 47)	(38/47 or 80.9%)	46.6% (n = 48)	(36/48 or 75.0%)	0.49
Shared decision	28.0% (n = 26)	(25/26 or 96.2%)	21.4% (n = 22)	(13/22 or 59.1%)	<0.01

* The P value represents the Chi-square test for reported receipt of mammography screening by age stratified by preferred role in decision-making. There is no significant difference by age in older women's preferred roles in decision making (p = 0.22).

†One woman was uncertain whether or not she had a mammogram in the past 2 years

## Discussion

In this study we identified factors, such as a doctor's recommendation, habit, and the need for reassurance, that are very important to older women when deciding to undergo mammography screening. Understanding the influence of these factors in older women's decision-making is important to improve counseling around mammography screening. Ideally, communication and decision-making would improve such that most women aged 80 and older in poor health and unlikely to benefit would choose not to undergo screening while most women aged 80 and older in good health would choose to undergo screening. In this study we found that a doctor's recommendation is the most highly ranked factor influencing older women's decision to get screened. Moreover, screening is common among women who prefer that they alone or their doctor makes the final decision whether or not they get screened regardless of health. In contrast, among women aged 80 and older who preferred to share decision-making around screening with their physicians, patient health plays a significant role in decision-making. This may indicate that when physicians have the opportunity to discuss the risks and benefits of mammography screening, and patients have an opportunity to discuss their values and perspectives, older women feel comfortable stopping screening. Interventions aimed at improving clinician counseling about mammography screening with older women, which include discussions around habit and reassurance, may result in more optimal decision-making.

Although several studies offer clinicians advice on how to encourage patients to undergo mammography screening [14-16] and two studies provide clinicians data to help determine which of their older patients may benefit from screening [1,17], we are unaware of studies that offer clinicians advice on how to discuss stopping screening with their patients. As it is important for clinicians to learn how to recommend screening, it is also important for clinicians to learn how to discuss stopping screening, especially since older patients report that they want to have these discussions with their clinicians [18]. When discussing stopping screening with elderly women in poor health, clinicians may want to acknowledge they understand how hard it must be for a patient to stop going for mammography when they have been doing so for years. Clinicians may also want to explain that just as there is a time to start screening, there is also a time to stop screening. Clinicians should discuss the risks of screening elderly women in poor health (e.g., unknown benefit of screening and complications from work-up and treatment of breast cancer). In addition, since a need for reassurance about one's health is important to patients, clinicians should be sure to offer patients reassurance by saying they will focus on preventive health measures (e.g., screening for geriatric health issues) that are more likely to benefit these women. This may help prevent patients from feeling like their doctor is giving up on them.

Although it is difficult to draw conclusions about factors that influence older women to choose not to be screened due to the low numbers of these women in our study, women aged 80 and older who chose not to be screened ranked age and then doctor's counseling as the most important factors influencing their decision. In fact, women aged 80 and older were significantly more likely than younger women to score age highly in their decision not to get screened. Resnick et al. also found that age and a lack of a doctor's recommendation were the most common reasons older adults living in a retirement community gave for not undergoing screening [19]. Physicians may need to explain to older women in good health who prefer not to be screened, that health rather than age should influence their decision. This is especially important since observational studies have found a mortality benefit of regular mammography screening for women aged 80–84 in good health [3,20].

We additionally found that shared decision-making around mammography screening between women aged 80 and older and their clinicians may result in better targeting of screening to older women in good health. Shared decision making occurs when clinicians involve patients as active partners in clarifying and choosing acceptable medical options [21]. Experts recommend that clinicians engage in shared decision making with patients when there is insufficient evidence about the risk-benefit ratio of a test; such as in the case of mammography screening among women aged 80 and older [21]. Interventions or tools, such as decision aids, aimed at improving shared decision-making around mammography screening between clinicians and their elderly patients may result in more optimal use of screening [22].

There are several limitations to this study. We interviewed English speaking women at one academic primary care practice in Boston and our results may not be generalizable to other women, especially since mammography screening was more common among elderly women in this study compared to national studies. However, the factors influencing older women's decisions to choose screening should be similar to other older women well connected to primary care and for whom counseling about screening is most likely to occur. Not all women eligible for the study chose to participate, however, non-respondents were similar to respondents with respect to age, race/ethnicity, illness burden, and previous mammography screening. In addition, our response rate is similar to other telephone surveys assessing older adults screening decisions [23,24]. Since few women in our practice chose not to get screened with mammography, we have less data on why women choose not to undergo mammography screening than on why women choose to undergo screening. Finally, Degner's decision-making preference scale may oversimplify women's thoughts about their preferred role in decision-making around mammography screening [25]. Despite these limitations, this study provides important information on factors that influence elderly women's mammography screening decisions that may guide future interventions.

## Conclusion

In summary, we found that several factors are highly important to older women's mammography screening decisions, including their doctor's recommendation, habit, and the need for reassurance; yet, personal health was not factored into these decisions. Physicians may want to address the influential roles of habit and reassurance and the unappreciated role of health to improve mammography screening discussions. Optimal decision-making may occur when elderly women have the opportunity to participate in shared decision-making around mammography screening with their clinicians.

## Competing interests

The author(s) declare that they have no competing interests.

## Authors' contributions

MS designed the study, participated in data collection, analyzed and interpreted the data, and prepared the manuscript. EP helped to design the study, analyze and interpret the data, and prepare the manuscript. MY collected the data and helped to prepare the manuscript. RD helped to design the study, analyze the data, and prepare the manuscript. EM helped to design the study, analyze and interpret the data, and prepare the manuscript.

## Pre-publication history

The pre-publication history for this paper can be accessed here:


